# Length of stay in pediatric intensive care unit: prediction model

**DOI:** 10.31744/einstein_journal/2020AO5476

**Published:** 2020-10-02

**Authors:** Simone Brandi, Eduardo Juan Troster, Mariana Lucas da Rocha Cunha

**Affiliations:** 1 Hospital Israelita Albert Einstein São PauloSP Brazil Hospital Israelita Albert Einstein, São Paulo, SP, Brazil.; 2 Faculdade Israelita de Ciências da Saúde Albert Einstein Hospital Israelita Albert Einstein São PauloSP Brazil Faculdade Israelita de Ciências da Saúde Albert Einstein, Hospital Israelita Albert Einstein, São Paulo, SP, Brazil.

**Keywords:** Lengh of stay, Critical care, Logistic models, Forecasting, Heath management, Beds/supply & distribution, Intensive care units, pediatric

## Abstract

**Objective:**

To propose a predictive model for the length of stay risk among children admitted to a pediatric intensive care unit based on demographic and clinical characteristics upon admission.

**Methods:**

This was a retrospective cohort study conducted at a private and general hospital located in the municipality of Sao Paulo, Brazil. We used internal validation procedures and obtained an area under ROC curve for the to build of the predictive model.

**Results:**

The mean hospital stay was 2 days. Predictive model resulted in a score that enabled the segmentation of hospital stay from 1 to 2 days, 3 to 4 days, and more than 4 days. The accuracy model from 3 to 4 days was 0.71 and model greater than 4 days was 0.69. The accuracy found for 3 to 4 days (65%) and greater than 4 days (66%) of hospital stay showed a chance of correctness, which was considering modest. Conclusion: Our results showed that low accuracy found in the predictive model did not enable the model to be exclusively adopted for decision-making or discharge planning. Predictive models of length of stay risk that consider variables of patients obtained only upon admission are limit, because they do not consider other characteristics present during hospitalization such as possible complications and adverse events, features that could impact negatively the accuracy of the proposed model.

## INTRODUCTION

Advances in knowledge and health science technology have improved survival and prognosis of seriously ill children at pediatric intensive care unit (PICU), as well as for children in chronical ill conditions at intensive care units. Pediatric intensive care unit can improve survival rates of seriously ill children, however, this unit requires expensive equipment, specialized and trained teams. For this reason, PICU requires a large amount of hospital resources and the efficient management of intensive care therapy bed is crucial, given that this is a limited resource.^(1-3)^

We define length of stay (LOS) as the period between date and time of intensive care unit (ICU) admission, and date and time of ICU discharge.^(4)^ The efficiency and quality of critical care are associated with LOS, and its control is important to promote safety and patient satisfaction, in addition to maintain financial viability of hospitals. The LOS is an indirect measurement used for resources use and financial performance of ICU care.^(3-5)^

Although LOS in PICU in England is statically lower than 3 days, the Brazilian National Supplementary Health Agency (NSHA) shows that the mean of LOS in the ICU in Brazil ranges from 7.4 to 9.9 days.^(6,7)^

Policies for hospital discharge or different types of practice and bed management can affect the LOS in case that it would not be managed in an effective manner, and, consequently, influenced negatively the health status of a patient (for example, in cases of delay to free up beds), or by increasing the risk of infections and complications due to longer LOS in pediatric ICU. Other possible recurrence is the non-availability of beds that can result in cancellation of elective surgical procedures, leading to periods of unnecessary waiting and wasting of ward beds.^(4,8,9)^

Major efforts have been made by researchers and managers at intensive care environment in an attempt to predict LOS of patients with mathematical programming models for regression and algorithms developed based on physiological, diagnostic, and demographic information. The goal of these models is to predict LOS risk of patients considering these information.^(8)^

The prediction LOS proposal is to support responsible professional for daily management of beds or for clinical cases and, possibility, to establish actions in clinical practice to reduce LOS to benefit patient safety and care quality. Predictions of LOS can be useful to evaluate the use of bed, estimate the need of available resources, and number of beds required.^(8)^

Models to predict in the ICU are still limited in the published literature, despite their relevance. Studies and tests for risk model prediction of based on clinical variable of patients and characteristic on admission can allow to create a tool to estimate LOS and improve placement of resources and long-term strategic planning of health institutions.

## OBJECTIVE

To propose model to predict hospital length of stay risk based on demographic and clinical features on admission for children at pediatric intensive care unit.

## METHODS

### Type of study

This was a retrospective cohort study including 1,815 consecutive admissions at the pediatric ICU within a period of 30 months in which data were collected from July 2012 to December 2014. The unit where the study was conducted has 15 beds and is located at the *Hospital Israelita Albert Einstein*, a private facility in the city of Sao Paulo, Brazil.

### Data collection

Data were retrieved from a database of the PICU that is update daily and includes demographic features, clinical conditions, and hospitalization. Independent variables of the study identified upon admission were age, sex, indication for admission (elective, urgency, or emergency), type of admission, outcome (transference, hospital discharge, discharge with homecare, or death), the use of mechanical ventilation, origin (pediatric unit, emergency unit, surgical center, pediatric outpatient unit, bone marrow transplantation or external), readmission within 48 hours, reason for admission (respiratory failure, sepsis, shock, post-operatory, liver failure, neurology, hemodynamic monitoring, post-event monitoring, other), Paediatric Logistic Organ Dysfunction (PELOD) score, and presence of venous access.

### Selection criteria

We included patients with different dates on admission and discharge. We excluded newborns with less than 36 gestational weeks.

### Ethical aspects

The study was approved by the Ethical and Research Committee in 2015, CAAE: 39646314.0.0000.0071, technical report # 1.299.819. This study follows the resolution 466/2012 of National Health Council.

### Data analysis and treatment

Length of stay mean was 2 days, for analysis of length of stay we considered the following intervals: 1 to 2 days, 3 or 4 days (RS 3-4) and greater than 4 days (RS>4).

To create and validate models for prediction of LOS risk of RS 3-4 days and RS>4 days, database were divided into two parts (2:1), and the greater portion was used for adjusting the model and the lower portion was used to internal validation of predictive model obtained.

To obtain predictor models, we adjusted equations of generalized estimation with binomial distribution, logistic function linking and structure of autoregressive correlation that contained patient’s identification to control dependence of several measures of the same individual.

Internal validation was conducted by obtaining of ROC curve and its area under estimated curve for adjusted data, and validation sample with the aim to asses description of obtained models.

## RESULTS

**Figure 1:**
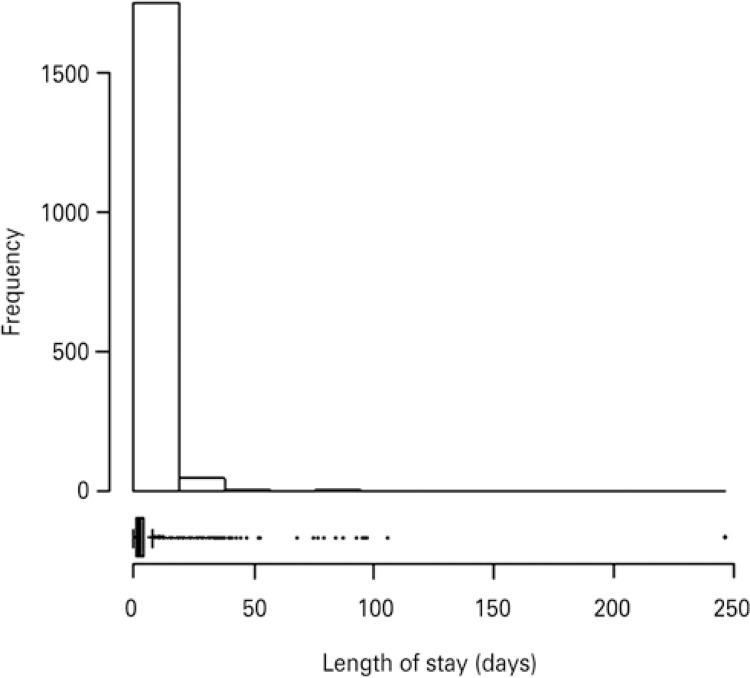
Distribution of frequency of length of stay of 1,815 admissions

Most of admitted patients had mean age of 19 months, and they had as the main indication of admission urgency and emergency condition (83.7%). Origin of children were predominantly from the emergency care (62.0%) due respiratory failure (38.9%). Readmission accounted for 0.7% of patients of the sample. We opted to use the mean of 2 days with first quartile, and third quartile of four days, once this distribution of data of LOS was not symmetrical (Figure 1). Therefore, we designated three segments for build of risk prediction model: 1 or 2 days, 3 or 4 days and greater than 4 days.

**Figure 2:**
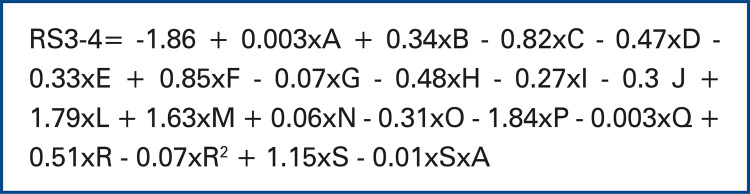
Formula for calculation of risk of length of stay from 3 to 4 days in the pediatric intensive care unit Legend A = Age in months; B = Male; C = Outpatient; D = Surgical Center; E = External; F = Pediatrics; G = Bone marrow transplant; H = Hemo-dynamic Monitoring; I = Post-event monitoring; J = Neurological; L = Large postoperative period; M = Medium-sized postoperative period; N = Small post-operative period; O = Sepsis; P = Trauma; Q = Other; R = PELOD score; S = Emergency/Urgency/Admission

To build the risk prediction model of LOS from 3 to 4 days in pediatric ICU, significant variables were: age in months, sex, origin, reason for admission, PELOD score and its square, indication for admission and interaction between indication for admission and age (in months). The formula for calculation of risk of LOS from 3 to 4 days in pediatric ICU is presented on figure 2.

**Figure 3:**
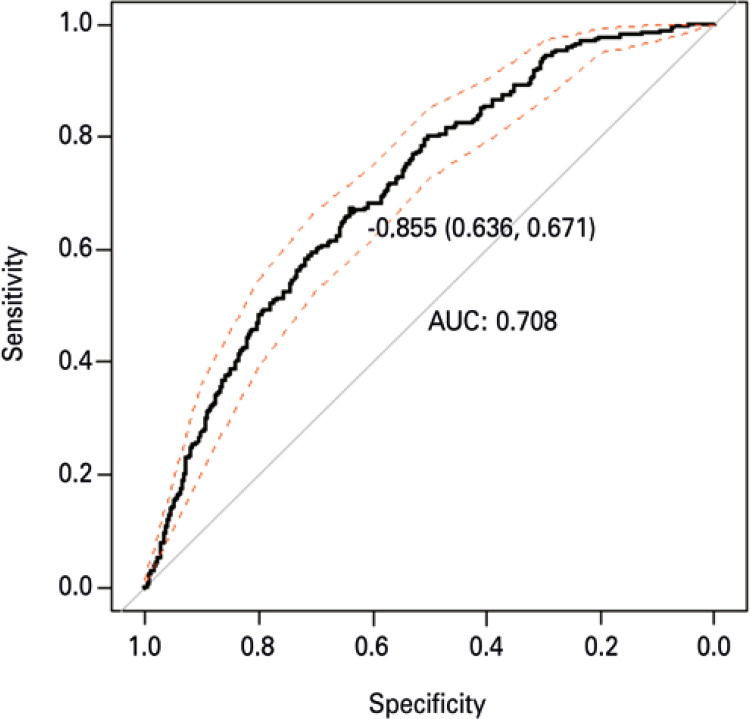
ROC curve for risk of hospitalization from 3 to 4 days obtained by multiple logistic model. The optimal cut-off point was indicated along with cut-off point (sensibility and specificity), n=912 AUC: area under the curve.

Predicted values for this model was constructed the ROC curve (Figure 3), and the area under the curve was 0.71. Predict values in ROC curve, upon admission, that presented greater than cut-off point (-0.855) indicated that risk would exist of LOS between 3 and 4 days. Lower predict values than the cut-off point (-0.855) would estimated risk between 1 and 2 days.

**Figure 4:**
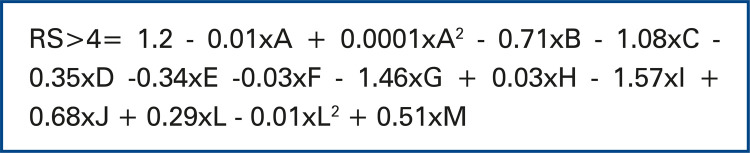
Form to calculate risk of length of stay greater than 4 days in the pediatric intensive care unit Legend A = Age in months; B = Hemo-dynamic monitoring; C = Post-event monitoring; D = Neurological; E = Large postoperative period; F = Medium-sized postoperative period; G = Small post-operative period; H = Sepsis; I = Trauma; J = Other; L = PELOD score; M = Venous Access

**Figure 5:**
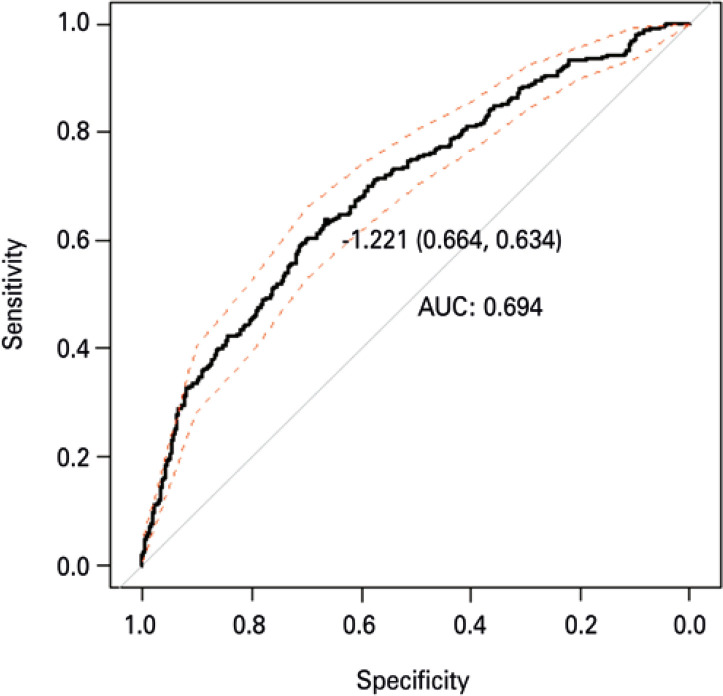
ROC curve for stay risk greater than 4 days obtained by the multiple logistic model. The cut-off point was indicated along with values of cut-off value (sensibility and specificity), n=1,197 AUC: area under the curve.

Similarly, in predictive for calculation of risk model with a LOS, more than 4 days, significant variables were age (in months), age-squared (in months), reasons for hospital admission, PELOD score and their square, and the use of venous access. The same model was adjusted to data, excluding different outliers, with LOS higher than 50 days and 30 days, and the sense of effects that did not change, neither conclusions about significance of each one of the variables included in the model, showing that predictive risk model did not suffer considerable influence of such disrupting outliers. The form to calculate risk of LOS greater than 4 days in pediatric ICU is presented in figure 4. The area under the AUC curve was 0.69 with cut-off point for risk score estimated from -1.221 (Figure 5).

Finally, to obtain the predictive score, first there was the need to use model of risk prediction for LOS greater than 4 days. Initially, we defined that stay would be lower or greater than 4 days. In case of score value they would be lower than -1.221 that should be applied the prediction model from 3 to 4 days, while, if the value of score would be lower than -0.855, the estimated stay would be 1 to 2 days. Finally, the value would be greater than -0.855, the LOS would be from 3 to 4 days.

## DISCUSSION

Severe ills patients who for the two decades would die, currently, are exposed to longer periods of hospitalization, late death and a varied degrees of disability, in order to prevent death. Advances in technology, family expectation, or health care team correspond to these changes. Therefore, the early detection of risk of hospital stay among hospitalized patient would help health care team and patients’ families to understand implications of the disease and the longer LOS. Potential results would also allow care planning, aiming, whenever possible, hospital discharge.^(1,2)^

Prediction models of risk are used to prevent risk of specific event – in this case, the chance of hospitalization from 1 to 2 days, 3 to 4 days, and greater than 4 days. In this sense, these models will only be useful if they brought results that help adequate planning for hospital discharge, avoid delays in hospital admissions that required intensive care, and favor the adequate management of physical and human resources.^(3)^

Hospital discharge on the adequate time, without risk for the patient, would also be a concern for the manager, with the reason to avoid early hospital discharge and readmission. A study that evaluated readmission on pediatric ICU reported that 62% of patients were readmitted due to worsening in primary condition. The prediction of LOS can optimize the sustainability of resources, but such prediction does not promote iatrogenic conditions in patient care.^(10)^

In this study, accuracy found for periods from 3 to 4 days and greater than 4 days of hospital stay showed a chance of correctness that was considered modest, from 65% and 66%, respectively. In this case, to adopt the model for individuals affected by clinical issues would require an excellent accuracy, *i.e.*, above 0.90 and, probably, the clinical judgment of care team would be more precise than the calculated risk for this model.

It was possible to identify that only the use of clinical data and, probably, some of characteristics of patient upon admission had limited prediction of LOS of patients admitted to pediatric ICU, showing a better discrimination and accuracy. One study that evaluated the implantation of project for participation of nurses on bedside clinical visits on pediatric ICU identified reduced LOS from 2.5 days before visits, to 2.10 days during the project. In addition, the average score on satisfaction surveys of hospitalized patients raised from 82.4 to 92.2%.^(11)^ The results of this specific study reinforce the importance take into account other factors for assessment of LOS.

There are other factors that influence in LOS of patients admitted to pediatric ICU such as complications due to clinical conditions, for example, pneumothorax, renal failure, neuromuscular and vasoactive infusions, and also associated bloodstream infection, which require sophisticated methods of life support and increase LOS and pediatric ICU-costs.^(3,12)^

A study that evaluated indications of care quality in pediatric ICU with seven beds, on northeast region of Brazil, pointed out for mean LOS from 15.52±0.94 days, and concluded that this mean would be related with profile of patients with diagnosis of CET hospitalized in the region. Still, the LOS was lower when the time for mechanical ventilation needed was also lower.^(13)^

Prediction risk models that support previous knowledge of LOS are important tools given the possibility of compare them with real time outcomes and conduct indirect assessment of care quality. In addition to supporting decision-making in bed management.^(8)^

Of note is that the use of LOS as quality indicator to assess services and to the fair control of hospital costs involve the risk of this approach leading to highly insafe practice, which is inappropriate and early approach. The objective of high-accurate discharge, *i.e.*, time for LOS prediction, is not to promote insecurity conditions for patient care process, but to encourage the adoption of the actions and programs that avoid complications associated with long hospital stay in pediatric ICU.^(8,9)^

Prediction risk models of LOS that consider only variables obtained from patient during admission are limited, particularly that these variables do not consider other characteristics during the hospitalization such as possible complications and adverse events that may be associated with increase of LOS in ICU.^(14)^ A study identified that only the use of characteristics of admission of patients and the use of physiological profile are not conclusive enough to estimate LOS of patients. There is a need to include therapeutic factors after admission to investigate this estimation and to propose multivariable models.^(3)^

We should consider, for example, that children with chronic health conditions often present previous disease (underlying disease) and require continuing healthcare. A recent study showed that children with chronic conditions have higher risk of long-term stay than those without these conditions. Therefore, not considering the assessment of prevalence and the type of chronic condition can lead to a barrier to predicting LOS for these children.^(1,2)^

However, to consider to include post-admission variables that would be possible to increase the chance of accurate prediction model would end up decrease the impact of advantages of immediate estimation of LOS upon admission.

The institutional factor, which was not evaluated in our study, is likely to be the cause of variability in LOS and limitation of the accurate prediction models. Institutional factors that can be mentioned are clinical protocols, end-of-life management practices and care philosophy adopted that influence significantly performance of statistical models. When these factors are not included in the calculation of the length of stay, the prediction models are not able to consider changes in intensive practice and in the use of therapeutic resources that occur during hospitalization.

## CONCLUSION

This study enabled to construct predictive risk model of length of stay of children upon admission at the pediatric intensive care unit by considering patients’ characteristics and clinical data. Demographic features, clinical conditions and hospitalization of patients on admission that presented best predictors of length of stay of 3-4 days at the pediatric intensive care unit were age (in months), sex, origin, reason for admission, and interaction between indication for admission and age (in months).

In the predictive model for more than 4 days at the pediatric intensive care unit significant variables were age (in months), age-squared (in months), reason for admission, Paediatric Logistic Organ Dysfunction score and its square, and the use of intravenous access.

Despite the low accuracy found that, in certain extent, limits the support to decision making of healthcare team in terms of resource management and planning of patient discharge. Our study contributes to extent the literature and advance the knowledge related to patients’ admission factors that interfere in length of stay at pediatric intensive care units.
