# Supplementary material for: Length of stay in pediatric intensive care unit: prediction model
Source: Einstein (Sao Paulo). 2020 Oct 2;18:eAO5476. doi: 10.31744/einstein_journal/2020AO5476 (PMC7531900; doi:10.31744/einstein_journal/2020AO5476)
Supplement: Translation

# Translation: Tempo de permanência em unidade de terapia intensiva pediátrica: modelo de predição

**Authors:** Simone Brandi, Eduardo Juan Troster, Mariana Lucas da Rocha Cunha

**Affiliations:** 1 Brasil Hospital Israelita Albert Einstein, São Paulo, SP, Brasil.; 2 Brasil Faculdade Israelita de Ciências da Saúde Albert Einstein, Hospital Israelita Albert Einstein, São Paulo, SP, Brasil.

**Keywords:** Tempo de permanência, Cuidados críticos, Modelos logísticos, Previsões, Gestão em saúde, Leitos/provisão e distribuição, Unidades de terapia intensiva pediátrica

## Abstract

**Objetivo:**

Propor um modelo de predição de risco de permanência das crianças na unidade de terapia intensiva pediátrica, considerando-se as características demográficas e clínicas na admissão.

**Métodos:**

Coorte retrospectiva realizada a partir da análise de 1.815 admissões na terapia intensiva pediátrica, em um hospital privado e geral, do município de São Paulo (SP). Foram utilizados procedimentos de validação interna e obtenção da área sob a curva ROC na construção do modelo preditor.

**Resultados:**

A mediana do tempo de permanência foi de 2 dias. O modelo preditor produziu um escore que permitiu a segmentação do tempo de permanência de 1 a 2 dias, de 3 a 4 dias e maior que 4 dias. A acurácia do modelo de 3 a 4 dias foi de 0,71 e do modelo maior que 4 dias de 0,69. As acurácias encontradas para 3 a 4 dias e maior que 4 dias de permanência mostraram possibilidade de acerto, considerada modesta, de 65% e 66%, respectivamente.

**Conclusão:**

A partir dos resultados encontrados, é possível verificar que a baixa acurácia encontrada no modelo preditor não permite que ele seja exclusivamente adotado para a tomada de decisão ou planejamento para a alta. Modelos de predição de risco do tempo de permanência que consideram variáveis do paciente obtidas somente durante a admissão têm limitações intrínsecas, já que não consideram outras características presentes durante a internação, como possíveis complicações e eventos adversos, e podem impactar negativamente na acurácia do modelo proposto.

## INTRODUÇÃO

Os avanços no conhecimento e na tecnologia na ciência da saúde trouxeram melhora da sobrevivência e do prognóstico da criança gravemente doente nas unidades de terapia intensiva (UTI) pediátricas, assim como das crianças com condições crônicas, que requerem cuidados intensivos. O cuidado em UTI pediátrica pode melhorar os índices de sobrevivência da criança gravemente enferma, mas necessita de equipamentos caros, equipe especializada e treinada. Assim, consome grande parte dos recursos dos hospitais, sendo importantes a efetividade e a eficiência do uso do leito de terapia intensiva, visto que se constitui em recurso quantitativamente limitado.^(1-3)^

Define-se tempo de permanência (TP) como o período entre a data e a hora da admissão na UTI e a data e a hora da alta da UTI.^(4)^ A eficiência e a qualidade do cuidado crítico estão associadas ao TP, e seu controle é importante para a promoção da segurança e da satisfação do paciente, além de fomentar a viabilidade financeira dos hospitais. O TP é uma medida indireta da utilização de recursos e do desempenho financeiro e assistencial na UTI.^(3-5)^

Apesar do TP estatisticamente observado nas UTI pediátricas inglesas, por exemplo, ser menor que 3 dias, os dados brasileiros da Agência Nacional de Saúde Suplementar (ANSS) mostram que o TP médio de permanência em UTI no contexto brasileiro é de 7,4 a 9,9 dias.^(6,7)^

Políticas de alta hospitalar ou diferentes tipos de prática e administração dos leitos podem afetar o TP, caso este não seja gerenciado de modo efetivo, e, consequentemente, influenciar negativamente no estado de saúde do paciente (nos casos de atraso na liberação dos leitos, por exemplo), ou aumentar o risco de infecções e complicações com o prolongamento do TP na UTI pediátrica. Outra possível decorrência é a indisponibilidade de leitos, que pode resultar em cancelamento de procedimentos cirúrgicos eletivos, levando a períodos de espera desnecessários e a desperdício de leitos na enfermaria.^(4,8,9)^

Há esforços, por parte de pesquisadores e gestores, no ambiente do cuidado intensivo, para tentar predizer o TP dos pacientes com a proposição de modelos matemáticos de regressão e algoritmos, que são desenvolvidos pautados por informações fisiológicas, diagnósticas e demográficas. O intuito desses modelos é a predição do risco de permanência do paciente, considerando estas especificidades.^(8)^

Nesse sentido, a proposta de predição do risco de permanência é a de auxiliar o profissional responsável pelo gerenciamento diário dos leitos ou de casos clínicos e, possivelmente, estabelecer ações na prática clínica para redução do TP, com benefícios para segurança do paciente e qualidade assistencial. A previsão da permanência pode ser útil para avaliar a utilização do leito, e estimar a necessidade de recursos disponíveis e o número de leitos requeridos.^(8)^

Modelos que possam predizer o TP em UTI ainda são limitados na literatura, apesar das indicações de sua importância. Assim, empenho em pesquisas na elaboração e na testagem de modelos de predição de risco de TP, com a utilização de variáveis clínicas do paciente e características na admissão, pode permitir a elaboração de uma ferramenta para estimar esse tempo e melhorar tanto a alocação de recursos, como o planejamento estratégico, a longo prazo, das instituições de saúde.

## OBJETIVO

Propor um modelo de predição de risco de permanência das crianças na unidade de terapia intensiva pediátrica.

## MÉTODOS

### Tipo do estudo

Trata-se de estudo de coorte retrospectiva de 1.815 admissões consecutivas na UTI pediátrica, referente a um período de 30 meses, cujos dados foram coletados no período de julho de 2012 a dezembro de 2014. A referida unidade possui 15 leitos e está locada no Hospital Israelita Albert Einstein, uma instituição privada da cidade de São Paulo.

### Coleta de dados

Os dados foram extraídos do banco de dados assistencial da UTI pediátrica, no qual são incluídas diariamente as informações de todos os pacientes, no que tange às características demográficas e às condições clínicas e de internação dos pacientes.

As variáveis independentes do estudo identificadas no momento da admissão foram idade, sexo, indicação de admissão (eletiva, urgência ou emergência), tipo de internação, desfecho (transferência, alta hospitalar, alta com *homecare* ou óbito), uso de ventilação mecânica, origem (pediatria, unidade de pronto atendimento – UPA –, centro cirúrgico, ambulatório de especialidades pediátricas, transplante de medula óssea ou externo), readmissão em 48 horas, motivo de admissão (insuficiência respiratória, sepse, choque, pós-operatórios, insuficiência hepática, neurológico, monitorização hemodinâmica, monitorização pós-evento, outros), escore de disfunção orgânica *Paediatric Logistic Organ Dysfunction* (PELOD) e presença de acesso venoso.

### Critérios de seleção

Foram incluídos pacientes com datas diferentes de admissão e alta. Foram excluídos recém-nascidos abaixo de 36 semanas gestacionais.

### Aspectos éticos

O estudo foi aprovado pelo Comitê de Ética da instituição do estudo, em 2015, sob CAAE: 39646314.0.0000.0071, parecer 1.299.819 e atendeu a resolução 466/2012 do Conselho Nacional de Saúde.

### Análise e tratamento dos dados

A mediana do TP foi de 2 dias, optando-se por analisar o risco de permanência considerando-se os seguintes intervalos: 1 a 2 dias, 3 ou 4 dias (ER 3-4) e maior que 4 dias (ER>4).

Para criação e validação de modelos de predição de risco de permanência de ER3-4 dias e ER>4 dias, o banco de dados foi dividido em duas partes (2:1), sendo a maior porção utilizada para o ajuste do modelo e a menor, para a validação interna do modelo preditor obtido.

Para a obtenção dos modelos preditores, foram ajustadas equações de estimação generalizadas, com distribuição binomial, função de ligação logística e estrutura de correlação autorregressiva, contendo a identificação do paciente para controle da dependência entre as diversas medidas de um mesmo indivíduo.

A validação interna foi conduzida por meio de obtenção da curva ROC e sua área sob a curva estimada para os dados ajustados e para a amostra da validação, com o intuito de avaliar a discriminação dos modelos obtidos.

## RESULTADOS

A maioria dos pacientes admitidos possuía a mediana de idade de 19 meses, tendo como principal indicação de admissão condição de urgência e emergência (83,7%). As crianças eram predominantemente oriundas da UPA (62,0%), por insuficiência respiratória (38,9%). As readmissões representam 0,7% dos pacientes da amostra.

Optou-se por utilizar a mediana de 2 dias, com primeiro quartil de 1 dia e terceiro quartil de quatro dias, uma vez que a distribuição dos dados de TP foi não simétrica (Figura 1). Assim, foram designados três segmentos para a construção do modelo de predição de risco: 1 ou 2 dias, 3 ou 4 dias e maior que 4 dias.

**Figura 1:** Distribuição de frequência do tempo de permanência das 1.815 admissões

Para construção do modelo de predição de risco do TP de 3 a 4 dias na UTI pediátrica, as variáveis significativas foram: idade em meses, sexo, origem, motivo de internação, escore PELOD e seu quadrado, indicação de admissão e interação entre indicação de admissão e idade (em meses). A fórmula para o cálculo do risco do TP de 3 a 4 dias na UTI pediátrica é apresentada na figura 2.

**Figura 2:** Fórmula para cálculo do risco do tempo de permanência de 3 a 4 dias na unidade de terapia intensiva pediátrica Legenda A = Idade em meses; B = Masculino; C = Ambulatório; D = Centro Cirúrgico; E = Externo; F = Pediatria; G = Transplante de medula óssea; H = Monitorização hemodinâmica; I = Monitorização pós evento; J = Neurológico; L = Pós operatório grande porte; M = Pós operatório médio porte; N = Pós operatório pequeno porte; O = Sepse; P = Trauma; Q = Outro; R = Escore PELOD; S = Admissão Urgência/Emergência

Com os valores preditos por esse modelo, foi construída a curva ROC (Figura 3), e a área sob a curva foi de 0,71. Os valores preditos na curva ROC, no momento da admissão, que se apresentassem maiores que o ponto de corte (-0,855), indicariam que existia risco de TP entre 3 e 4 dias. Valores preditos menores que o ponto de corte (-0,855) estimariam risco entre 1 a 2 dias.

**Figura 3:** Curva ROC para o risco de permanência 3 a 4 dias obtida pelo modelo logístico múltiplo. O ponto de corte ótimo foi indicado junto ao valor do ponto de corte (sensibilidade e especificidade), com n=912 AUC: área sob a curva.

Da mesma forma, no cálculo para o modelo de predição de risco de permanência maior que 4 dias, as variáveis significativas foram idade (em meses), idade ao quadrado (em meses), motivo de admissão, escore PELOD e seu quadrado, e uso de acesso venoso. O mesmo modelo foi ajustado aos dados, excluindo-se valores discrepantes, com TP superior a 50 dias e superior a 30 dias, e o sentido dos efeitos não se alterou, nem as conclusões sobre a significância de cada uma das variáveis incluída no modelo, evidenciando-se que o modelo de predição de risco não sofreu influência considerável de tais valores discrepantes. A fórmula para o cálculo do risco do TP maior que 4 dias na UTI pediátrica é apresentada na figura 4. A área sob a curva ROC foi de 0,69, com ponto de corte para o escore de risco estimado de -1,221 (Figura 5).

**Figura 4:** Fórmula para cálculo do risco do tempo de permanência maior que 4 dias na unidade de terapia intensiva pediátrica Legenda A = Idade em meses; B = Monitorização hemodinâmica; C = Monitorização pós evento; D = Neurológico; E = Pós operatório grande porte; F = Pós operatório médio porte; G= Pós operatório pequeno porte; H = Sepse; I = Trauma; J = Outro; L = Escore PELOD; M = Acesso Venoso

**Figura 5:** Curva ROC para risco permanência maior que 4 dias obtida pelo modelo logístico múltiplo. O ponto de corte ótimo foi indicado junto ao valor do ponto de corte (sensibilidade e especificidade), com n=1.197 AUC: área sob a curva.

Para obtenção do escore preditivo, foi necessário, primeiramente, utilizar o modelo de predição de risco para TP maior que 4 dias. Isto, inicialmente, definiu se a permanência seria menor ou maior que 4 dias. Caso o valor do escore fosse menor que -1,221 deveria ser aplicado o modelo de predição de 3 a 4 dias, enquanto que, se o valor do escore fosse menor que -0,855, a permanência estimada seria de 1 a 2 dias. Finalmente, se o valor fosse maior do que -0,855, o TP seria de 3 a 4 dias.

## DISCUSSÃO

Os pacientes graves que há duas décadas morreriam, atualmente, são expostos às internações prolongadas, mortalidade tardia e graus variados de incapacidade, a fim de se tentar evitar o desfecho de óbito. Avanços na tecnologia e expectativa da família ou da equipe assistencial correspondem a essas mudanças. Assim, a identificação precoce do risco de permanência dos pacientes ajudaria a equipe de saúde e as famílias a entenderem as implicações da doença e do TP prolongado. Os potenciais resultados permitiriam ainda o planejamento dos cuidados, visando, sempre que possível, à desospitalização do paciente.^(1,2)^

Os modelos de predição de risco são utilizados para prever o risco de um evento específico – neste caso, a chance de permanência 1 a 2 dias, 3 a 4 dias e maior que 4 dias. Nesse sentido, somente serão úteis se trouxerem resultados que auxiliem o planejamento adequado da desospitalização; evitarem atrasos nas admissões de pacientes que requerem cuidado intensivo; e favorecerem a gestão adequada de recursos humanos e físicos.^(3)^

A desospitalização em tempo adequado, sem riscos para o paciente, também deve ser preocupação do gestor, com o intuito de evitar alta precoce e readmissão. Uma pesquisa que avaliou a readmissão em UTI pediátrica detectou que 62% dos pacientes foram readmitidos devido à piora da condição primária. A predição do TP pode otimizar a sustentabilidade dos recursos, mas não deve promover condições iatrogênicas no cuidado ao paciente.^(10)^

No presente estudo, as acurácias encontradas para 3 a 4 dias e maior que 4 dias de permanência mostraram uma possibilidade de acerto considerada modesta, de 65% e 66%, respectivamente. Neste caso, para a adoção do modelo no caso de indivíduos impactados por questões clínicas, seria necessária uma acurácia excepcional acima de 0,90 e, provavelmente, o julgamento clínico da equipe assistencial poderia ser mais preciso que o risco calculado por este modelo.

Foi possível identificar que somente a utilização de dados clínicos e, provavelmente, de algumas das características do paciente na admissão limitou a predição do TP dos pacientes admitidos na UTI pediátrica, com melhor discriminação e acurácia. Um estudo que avaliou a implantação de um projeto para participação dos enfermeiros nas visitas clínicas à beira do leito em UTI pediátrica identificou diminuição do TP de 2,5 dias, antes das visitas, para 2,10 dias, durante o projeto. Além disto, as pontuações nas pesquisas de satisfação de pacientes internados aumentaram de 82,4 para 92,2%.^(11)^ Os resultados deste referido estudo reforçam a importância de se considerar outros fatores para a avaliação do TP.

Há outros fatores que influenciam no TP do paciente admitido em uma UTI pediátrica, como as complicações decorrentes das condições clínicas, como pneumotórax, falência renal, bloqueio neuromuscular e infusões vasoativas, e também as taxas de infecção associada à corrente sanguínea, que exigem métodos sofisticados de suporte à vida e aumentam o TP e os custos nas UTI pediátricas.^(3,12)^

Estudo que avaliou os indicadores de qualidade assistencial em uma UTI pediátrica de sete leitos, na Região Nordeste do país apontou para um TP médio de permanência de 15,52±0,94 dias e concluiu que esta média teria relação com o perfil de pacientes com diagnóstico de traumatismo craniencefálico internados na região. Ainda, o TP foi menor quando o tempo de ventilação mecânica necessário também fora menor.^(13)^

Modelos de predição de risco que auxiliem no conhecimento prévio do TP são ferramentas importantes, dada a possibilidade de comparação com o desfecho real de tempo e por permitirem a avaliação indireta da qualidade do cuidado, além da previsão de permanência apoiar a tomada de decisão no gerenciamento do leito.^(8)^

Todavia, ao utilizarmos o TP como indicador de qualidade para avaliação do serviço e controle satisfatório dos custos hospitalares, há um risco dessa abordagem conduzir à prática de alta insegura, que é inapropriada e precoce. O objetivo da previsão acurada da alta, ou seja, de predizer o TP, não é acrescentar condições inseguras ao processo do cuidado do paciente, mas encorajar a implantação de programas que evitem as complicações associadas à longa permanência nas UTI pediátricas.^(8,9)^

Modelos de predição de risco do TP que consideram variáveis do paciente obtidas somente durante a admissão têm limitações intrínsecas, já que não consideram outras características presentes durante a internação como possíveis complicações e eventos adversos, que mostraram-se associados ao aumento do TP na UTI.^(14)^ Estudo identificou que somente a utilização de características da admissão dos pacientes e o uso do perfil fisiológico não são suficientemente conclusivos para estimar o TP dos pacientes, e a inclusão de fatores terapêuticos pós-admissão é importante na investigação desta relação, para a proposição dos modelos multivariáveis.^(3)^

Há que se considerar, por exemplo, que crianças com condições de saúde crônicas possuem doenças prévias (de base) com necessidades contínuas de cuidados. Estudo recente mostra que as condições crônicas presentes nas crianças aumentam seu risco de permanência em relação às outras sem essa condição. Portanto, não ponderar sobre a prevalência e o tipo de condição crônica dificulta a predição do TP dessas crianças.^(1,2)^

No entanto, ao se considerar incluir as variáveis pós-admissionais, que possivelmente aumentariam a chance de acerto do modelo de predição, acarretaria em desvalorizar as vantagens de se ter uma estimativa de TP imediata ao momento da admissão.

O fator institucional é provavelmente causa da variabilidade no TP e, quando não avaliado, como neste estudo, limita a acurácia dos modelos de predição. Entre os fatores institucionais que podem ser citados, destacam-se os protocolos clínicos, as práticas de manejo de final de vida e os eventos ou intercorrências clínicas que ocorrem durante a internação e podem influenciar significativamente nos desfechos clínicos nas UTI pediátrica e, consequentemente no desempenho dos modelos estatísticos. Quando estes fatores não são incluidos no cálculo de estimativas de tempo de permanência, os modelos de predição não são capazes de considerar as mudanças na prática intensiva e na utilização dos recursos terapêuticos que ocorrem ao longo da internação.

## CONCLUSÃO

Este estudo possibilitou a construção de um modelo de predição de risco de tempo de permanência das crianças no momento da admissão na unidade de terapia intensiva pediátrica, considerando-se as características e os dados clínicos dos pacientes.

As características demográficas, e as condições clínicas e de internação dos pacientes na admissão que se apresentaram como melhores preditores do tempo de permanência de 3 a 4 dias na unidade de terapia intensiva pediátrica foram idade (em meses), sexo, origem, motivo de admissão, escore PELOD e seu quadrado, indicação de admissão e interação entre a indicação de admissão e idade (em meses). Considerando-se o modelo preditor para mais de 4 dias na unidade de terapia intensiva pediátrica, foram significativas as variáveis idade (em meses), idade ao quadrado (em meses), motivo de admissão, escore PELOD e seu quadrado, e uso de acesso venoso.

Apesar da baixa acurácia encontrada, que, de certa forma, limita o apoio à tomada de decisão da equipe assistencial para a gestão dos recursos e planejamento da alta do paciente, evidencia-se que o estudo avançou no conhecimento referente aos fatores admissionais do paciente, que interferem no tempo de permanência na unidade de terapia intensiva pediátrica e colabora com a literatura sobre o tema.
